# Molecular Crowding Inhibits U-Insertion/Deletion RNA Editing *In Vitro*: Consequences for the *In Vivo* Reaction

**DOI:** 10.1371/journal.pone.0083796

**Published:** 2013-12-23

**Authors:** Venkata Subbaraju Katari, Lea van Esdonk, H. Ulrich Göringer

**Affiliations:** Department of Molecular Genetics, Darmstadt University of Technology, Darmstadt, Germany; University of Florida, United States of America

## Abstract

Mitochondrial pre-mRNAs in African trypanosomes are edited to generate functional transcripts. The reaction is typified by the insertion and deletion of U nucleotides and is catalyzed by a macromolecular complex, the editosome. Editosomes bind pre-edited mRNA/gRNA pairs and the reaction can be recapitulated *in vitro* by using pre-mRNA- and gRNA-mimicking oligoribonucleotides together with enriched editosome preparations. Although the *in vitro* assay has been instrumental in unraveling the basic steps of the editing cycle it is performed at dilute solvent conditions. This ignores the fact that editing takes place inside the highly crowded mitochondria. Here we investigate the effects of molecular crowding on RNA editing. By using neutral, macromolecular cosolutes we generate defined dilute, semidilute and crowded solvent properties and we demonstrate different thermodynamic stabilities of the pre-mRNA/gRNA hybrid RNAs at these conditions. Crowded conditions stabilize the RNAs by -30 kJ/mol. Furthermore, we show that the rate constants for the association and dissociation (k_ass_/k_diss_) of substrate RNAs to editosomes decrease, ultimately inhibiting the *in vitro* reaction. The data demonstrate that the current RNA editing *in vitro* system is sensitive to molecular crowding, which suggests that the *in vivo* reaction cannot rely on a diffusion-controlled, collision-based mechanism. Possible non-diffusional reaction pathways are discussed.

## Introduction

Chemical reactions in living systems take place in aqueous solutions that contain high concentrations of macromolecules. Intracellular concentrations can reach up to 400 g/L thereby generating “crowded” or “volume-occupied” solvent conditions [1]–[3]. Although no individual macromolecular species is present at a high concentration, together all macromolecules can occupy up to 30% of the total cell volume and thus, physically occupy a significant fraction of the cell [4]. In general, macromolecular crowding enhances biomolecular interactions and reactions that ultimately cause a reduction of the total excluded volume. This includes the formation of macromolecular complexes, the binding of macromolecules to surface sites as well as aggregation and folding/unfolding phenomena of nucleic acids and proteins [2]. Furthermore, volume exclusion affects the equilibrium and kinetics of macromolecular reactions with two opposing effects: while it increases the rate of slow, transition-state-limited association reactions, it decreases the rate of fast, diffusion-limited association reactions [1], [2].

**Figure 1:**
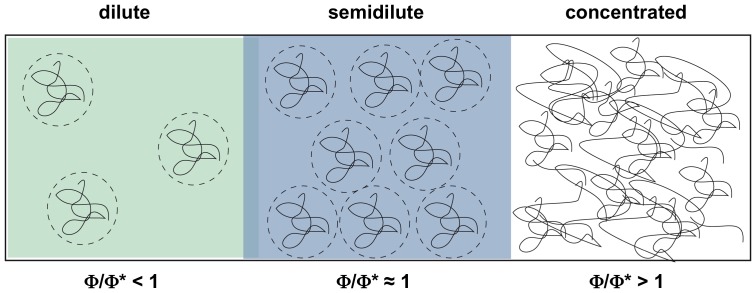
Schematic representation of dilute, semidilute and crowded cosolute properties (Wang et. al. 2010 [9]). Crowding reagents such as PEG or dextran can be approximated as elastic, coiled spheres (dashed circles). The polymers change their behavior in solution as a function of concentration. The character of the polymer-induced interaction changes significantly as one goes from dilute (green) to semidilute (blue) to crowded conditions (white). Dilute conditions (Φ<Φ*) are characterized by polymer concentrations (Φ) below the critical crossover concentration (Φ*) and thus the cosolute molecules are well separated from each other. In the semidilute regime (Φ≈Φ*) the polymers start to overlap and form network-like structures. At crowded conditions (Φ>Φ*) the polymer density is very high and the molecules become intricately entangled. For linear polymers, Φ* can be approximated as Φ* = N^−4/5^ [6], [7].

Volume-occupied solvent conditions can be generated *in vitro* by using high concentrations of chemically neutral, macromolecular cosolutes such as polyethylene glycol (PEG), Ficoll, dextran or bovine serum albumin (BSA) [1], [5]. The different compounds can be used to generate dilute, semidilute as well as crowded solvent properties depending on their “crossover polymer concentration” (Φ*) [6]–[8]. Φ* is a function of the number of monomers per polymer (N) (Φ* = N^−4/5^) and it represents the concentration (in w/w %) at which the polymer molecules start to form porous, network-like structures. At dilute conditions (Φ<Φ*), the polymers can be viewed as flexible, coiled spheres with a defined radius of gyration (R_g_). At semidilute conditions (Φ≈Φ*), the coils begin to overlap forming random networks with a mean mesh size ξ. ξ is a function of the polymer concentration (Φ) (ξ≈Φ^−3/4^) [8] and a further increase of Φ generates crowded solvent conditions (Φ>Φ*), which are characterized by a dense entanglement and interpenetration of the polymer coils [9] (Fig. 1).

Despite the fact that macromolecular crowding has been shown to impact a large number of biological processes involving proteins and protein complexes [10]–[12], its effect on nucleic acids especially on the structure, stability and function of RNA molecules is less studied. Multiple attributes of a crowded solution can affect the equilibrium between a properly folded, functional RNA and its unfolded, nonfunctional conformation(s). This includes a change in the chemical potential of the RNA due to a reduction of the available volume. The degree of volume exclusion is a consequence of the size of all macromolecules in the solution and, depending on the number of interactions, it is highly nonlinear with concentration [13]. In addition, crowding can impact the activities of ions in the solution thereby modulating one of the dominating forces of macromolecular folding. While small molecule osmolytes have been shown to destabilize RNA secondary structure and in some cases RNA 3D-structure due to unfavorable surface interactions [14]–[16], high molecular mass crowding reagents stabilize folded RNA (and DNA) conformations entropically due to the excluded volume effect [17]–[19]. This holds also true for large ribonucleoprotein (RNP) complexes such as ribosomes: the association of the two ribosomal subunits can be stimulated by chemically inert cosolutes [20]. Furthermore, the catalytic activity of the hammerhead ribozyme is enhanced in the presence of crowding reagents [21]–[23] as is the hairpin/pseudoknot transition of the human telomerase RNA [24]. Similarly, the formation of DNA (and perhaps RNA) three-way junctions (TWJ) [25], [26], of G-quadruplex structures [27], [28] and of DNA triple helices [29] are favored in crowded solutions. Next to the excluded volume effect, hydration has been identified as a crucial factor for the stability of RNA molecules in crowded solutions with opposite effects on the stabilities of RNA tertiary and secondary structures [23], [30], [31]. Neutral cosolutes can stabilize the water release reaction of RNA 3D-folds while at the same time disfavor the water-uptake reaction of Watson-Crick base pairs [23].

RNA editing describes a post-transcriptional modification reaction of mitochondrial pre-mRNAs that is characterized by the site-specific insertion and deletion of exclusively U nucleotides (nts) [32]. The reaction takes place within the single mitochondrion of trypanosomes, which represents the most “crowded” intracellular environment of eukaryotic cells. Intra-mitochondrial macromolecular concentrations can reach up to 560 g/L [33], [34]. Editing is catalyzed by a macromolecular machinery, the 20 S editosome [35]. The multienzyme complex has a calculated molecular mass of 0.8MDa and has been visualized by cryo-electron microscopy (EM) and atomic force microscopy (AFM) [36], [37]. Key players in the reaction are a specific class of small, non-coding RNAs known as guide (g)RNAs. gRNAs function as templates in the reaction. They basepair to cognate pre-edited mRNAs and dictate the number of U nts to be inserted and/or deleted by way of their primary sequence. Editosomes have a single substrate RNA binding site, which binds the two RNA species with nanomolar affinity [37]. The catalytic conversion takes place within a multifunctional reaction center that executes several enzyme activities: endo/exo-nuclease, terminal uridylyl transferase, RNA ligase and perhaps nucleotidyl phosphatase [32], [35]. Thus, the reaction likely requires several dynamic adjustments not only of the RNA substrate molecules [37] but also of the catalytic machinery itself.

Our current understanding of the editing reaction mechanism is derived from an *in vitro* assay system that relies on truncated, cognate pairs of synthetic, pre-edited mRNAs and gRNAs together with enriched 20 S editosome preparations [38]. The complexes are isolated from non-ionic detergent lysates of *Trypanosoma brucei* mitochondria [39] and the assay depends on the diffusion/collision-based interaction of the RNA reactants with the catalytic machinery. Since the *in vitro* reaction is capable of monitoring the formation of reaction intermediates, side products as well as fully edited reaction products, it has been instrumental in unraveling the individual steps of the reaction cycle. However, at the same time the assay is characterized by a number of inexplicable limitations. This includes the questions whether the reaction is diffusionally or transition-state controlled and whether the catalytic machinery acts processively or distributively. Although a single gRNA is able to edit several editing sites *in vivo*, *in vitro* only a single site can be converted. Also, while most mitochondrial pre-mRNAs require the successive action of multiple gRNAs, *in vitro* the action of only one gRNA can be addressed. One obvious inadequacy of the assay is that it is carried out at dilute solvent conditions, which differ significantly from the above-described “crowded” *in vivo* situation. Here we ask the question whether editing is affected by volume-occupied solvent conditions. We use neutral macromolecular copolymers to generate defined dilute, semidilute and crowded solvent conditions and examine three different aspects of the editing reaction: First, we analyze the thermodynamic stability of synthetic gRNA/pre-mRNA substrate RNAs at volume-occupied solvent conditions; second, we monitor the kinetic and thermodynamic characteristics of the binding reaction of 20 S editosomes to substrate gRNA/pre-mRNA hybrid RNAs and third, we measure the catalytic conversion of pre-edited mRNAs to edited RNAs in crowded solutions.

## Materials and Methods

### Crowding agents

The following crowding reagents were used: polyethylene glycol (PEG)200, PEG300, PEG400, PEG2000, PEG4000 as well as Ficoll400, Dextran150 and bovine serum albumin (BSA). Relevant physical parameters of the different compounds are listed in Table 1: molecular mass distribution, number of monomers/polymer (N), crossover polymer concentration (Φ*), polymer length/persistence length ratio (L/Lp) and viscosity (η). Depending on the individual Φ*-values the reagents were used to generate dilute (Φ<Φ*), semidilute (Φ≈Φ*) and crowded solvent conditions (Φ>Φ*) covering a Φ/Φ* range of 0–4.9.

**Table 1:** Relevant physical parameters of the different molecular crowding reagents.

crowding reagent	mol. mass distribution	conc. range tested	*N*	Φ*	*L/L_P_*	η range
	(g/mol)	% (w/v)		% (w/v)		(cSt)
PEG200	180–220	0.1–30	3.2	39	1.3	21–25
PEG300	270–330	0.1–30	5	28	2	31–35
PEG400	370–430	0.1–30	7	23	3	40–45
PEG2000	1810–2200	0.01–20	32	6	13	150–210
PEG4000	3740–4480	0.01–20	65	4	26	260–360
Ficoll400	300000–500000	0.001–10	1170	0.35	-	-
Dextran150	125000–175000	0.001–10	833	0.46	-	-
BSA	65000	0.001–10	609	0.6	-	-

Number of monomers/polymer (N), crossover polymer concentration (Φ*), polymer length/persistence length (L/Lp) and viscosity (η).

### Oligoribonucleotide synthesis and radioactive labeling

RNA oligonucleotides were synthesized by automated solid phase phosphoramidite chemistry using 2′-O-triisopropylsilyoxymethyl (TOM) protected phosphoramidites (synthesis scale 50nmoles). Purified RNA oligonucleotides were dissolved in 10 mM Tris/HCl pH 7.5, 1 mM EDTA and stored at −20°C. Concentrations were determined by UV absorbance measurements at 260 nm. The following sequences were synthesized: Insertion RNA editing - 5′CL18: GGAA-GUAUGAGACGUAGG, 3′CL13: AUUGGAGUUAUAG, gRNA_ins_: CUAUAACUCCGAUAAACC-UACGUCUCAUACUUCC. Deletion RNA editing - 5′CL22: GGAAAGGGAAAGUUGUGAUUUU, 3′CL15: GCGAGUUAUAGAAUA, gRNA_del_: GGUUCUAUAACUCGCUCACAACUUUCCCU-UUCC. RNAs were 5′ [^32^P]-labeled using T4 polynucleotide kinase (10U) and γ-[^32^P]-ATP (specific activity: 3000 Ci/mmol) as a substrate. A typical reaction contained 50pmol RNA and 50 µCi γ-[^32^P]-ATP in 50 mM Tris/HCl pH 7.6, 10 mM MgCl_2_, 5 mM DTT and was incubated at 37°C for 90 min. Radioactively labeled RNAs were purified in 12% (w/v) denaturing polyacrylamide gels followed by gel excision, gel extraction and ethanol precipitation.

### Editosome enrichment

Insect-stage *Trypanosoma brucei* cells of strain Lister 427 [40] were propagated in SDM-79 medium [41]. Ten litre cultures were grown to late log phase equivalent to a cell density of 1×10^7^ cells/mL. Cells were disrupted at isotonic conditions by N_2_-cavitation [42] and mitochondrial (mt) vesicles were isolated by differential centrifugation. Detergent lysates of the mt-vesicles were generated by incubation with 1% (v/v) Triton X-100 (2× critical micelle concentration (CMC)) in editing buffer (EB: 20 mM HEPES/KOH pH 7.5, 30 mM KCl, 10 mM Mg(OAc)_2_) containing 1 mM DTT, 1 mM PMSF, 1 µg/mL leupeptin and 10 µg/mL trypsin inhibitor. Editosomes were enriched by isokinetic ultracentrifugation in linear 10–35% (v/v) glycerol gradients [43] and fractionated. Editosome-containing fractions (app. S-value: 20–24 S; refractive indices 1.355–1.360) were pooled. Protein concentrations varied between 0.15–0.2 mg/mL. Samples were frozen in liquid N_2_ and stored at −20°C.

### *In vitro* RNA editing

*In vitro* RNA editing assays were performed as in Igo et al., 2000 and Igo et al., 2002 [48], [49] using [^32^P]-labeled substrate RNAs (specific activity: 8×10^5 ^cpm/pmol). Cognate gRNAs and mRNAs were annealed by heating at 70°C for 5 min and cooling to 25°C at a rate of 1°C/min. Reactions were performed using 0.5 µg enriched 20 S editosomes with 100 fmol of annealed substrate RNAs, 0.2 mM DTT, 0.5 mM ATP and 40 µM UTP (for insertion assay only) in EB at 27°C for 2 h. Edited RNAs were resolved in 18% (w/v) polyacrylamide gels containing 8 M urea, visualized by phosphorimaging and analyzed densitometrically. RNA editing activities (EA) were normalized to the activity in the absence of crowding reagent (Φ/Φ* = 0) and plotted as a function of the molecular crowder (MC) concentration (log_EA_ = f(conc_MC_)).

### UV hyperchromicity measurements

Absorbance versus temperature profiles (melting curves) of RNA substrates were recorded at 260 nm using a thermoelectrically controlled UV-spectrophotometer in 50 mM sodium cacodylate pH 6.5, 150 mM NaCl and 2 mM MgCl_2_. Measurements were performed in the presence of low and high molecular mass PEGs (PEG200, PEG300, PEG400, PEG2000, PEG4000) at Φ/Φ* ratios of 0.3–4.9 (Table 2). The temperature was scanned at a heating rate of 1°C/min at temperatures between 20°C and 90°C. Absorbance values were recorded with an average time of 0.5 s and data were collected every 0.1°C. T_m_-values were determined from derivative plots of absorbance *versus* temperature dA_260_/dT = f(T) and the half maximum of fraction folded (α) *versus* temperature plots generated by correcting the melting curves for upper and lower baselines [44]. ΔH and ΔS-values were determined from van't Hoff plots of ln(*K*) *versus* 1000/T(K) with the slope representing -ΔH/R and the y-intercept ΔS/R. ΔG was determined by ΔG = ΔH-TΔS = RTxlnK.

**Table 2:** Melting temperatures and thermodynamic parameters.

	%	Φ/Φ*	T_m_-1	ΔT_m_-1	T_m_-2	ΔT_m_-2	ΔG	ΔH	ΔS	ΔΔG
	(w/v)		(°C)	(°C)	(°C)	(°C)	(kJ/mol)	(kJ/mol)	(J/mol/K)	(kJ/mol)
**U-insertion**										
**w/o PEG**	**-**	0	54.0	**-**	77.0	**-**	−293.4	−1103	−2717	-
**PEG4000**	20	4.9	55.9	1.9	79.5	2.5	−324.8	−1482.3	−3879	−31.4
**PEG2000**	20	3.3	55.7	1.7	79.5	2.5	−322.9	−1472.8	−3857.3	−29.4
**PEG400**	30	1.1	50.2	−3.8	75.8	−1.2	−265.7	−926.4	−2220	27.7
	15	0.5	53.0	−1.0	76.5	−0.5	−279.7	−980.9	−2325	13.8
**PEG300**	30	0.8	48.6	−5.4	73.8	−3.2	−248.9	−880.5	−2118	44.5
	15	0.4	52.1	−1.9	75.8	−1.2	−268.9	−988.1	−2412	24.5
**PEG200**	30	0.6	46.7	−7.3	71.7	−5.3	−241.4	−842	−2014	51.7
	15	0.3	50.5	−3.5	74.4	−2.6	−252.3	−886.8	−2130	41.2
**U-deletion**										
**w/o PEG**	**-**	0	66.6	**-**	74.0	**-**	−283.8	−969.1	−2303.2	-
**PEG4000**	20	4.9	69.9	3.3	76.7	2.7	−317.4	−1189.8	−2593.3	−33.7
**PEG2000**	20	3.3	69.9	3.3	76.2	2.2	−315.4	−1192.6	−2943.1	−31.6
**PEG400**	30	1.1	65.4	−1.2	71.6	−2.4	−252.3	−762.7	−1715.1	31.4
	15	0.5	66.3	−0.3	73.2	−0.8	−255.1	−754.8	−1678.8	28.7
**PEG300**	30	0.8	63.2	−3.4	69.3	−4.7	−231.8	−627.7	−1334.2	51.9
	15	0.4	64.8	−1.8	72.0	−2.0	−250.9	−787.3	−1799.1	32.8
**PEG200**	30	0.6	60.3	−6.3	67.1	−6.9	−223.7	−625.8	−1359.3	60
	15	0.3	63.5	−3.1	70.5	−3.5	−230.3	−588.2	−1228.2	53.5

ΔG, ΔH and ΔS for the helix/coil transition of U-insertion and U-deletion mRNA/gRNA hybrid RNAs in the presence of high and low molecular mass PEGs at dilute, semi-dilute and crowded solvent conditions (Φ/Φ* varies from 0 to 4.9).

### Surface plasmon resonance (SPR) measurements

Guide RNAs were 3′-oxidized at 4°C in the dark in 50 mM NaOAc pH 4.8, 10 mM MgCl_2_, 100 mM NaCl and 10 mM NaIO_4_ [45]. Samples were desalted and ethanol precipitated. Oxidized gRNAs were covalently attached to the surface of an amino silane-derivatized microcuvette in 50 mM NaBH_3_CN in a buffer containing 100 mM Na_x_H_y_PO_4_ pH 7, 150 mM NaCl for 3 h at 27°C. Coupled gRNAs were annealed to pre-mRNAs for 5 min in EB to generate gRNA/pre-mRNA hybrid RNAs. Binding of 20 S editosomes to the gRNA/pre-mRNA hybrids was monitored in real time in the presence of 25% (w/v) PEG400 (Φ/Φ* = 0.9) and 20% (w/v) PEG2000 (Φ/Φ* = 3.3) as a shift in the resonant angle. k_diss_ and k_ass_ values were determined by plotting observed on rates (k_on(obs)_) as a function of the editosome complex concentration (k_on(obs)_ = k_ass_×[complex]+k_diss_). Equilibrium dissociation constants (K_d_) were calculated as K_d_ = k_diss_/k_ass_. Half-lives of 20 S editosome/RNA complexes were determined as t_1/2_ = ln2/k_diss_.

## Results

### Stability of gRNA/pre-mRNA hybrid RNAs at molecular crowding conditions

**Figure 2:**
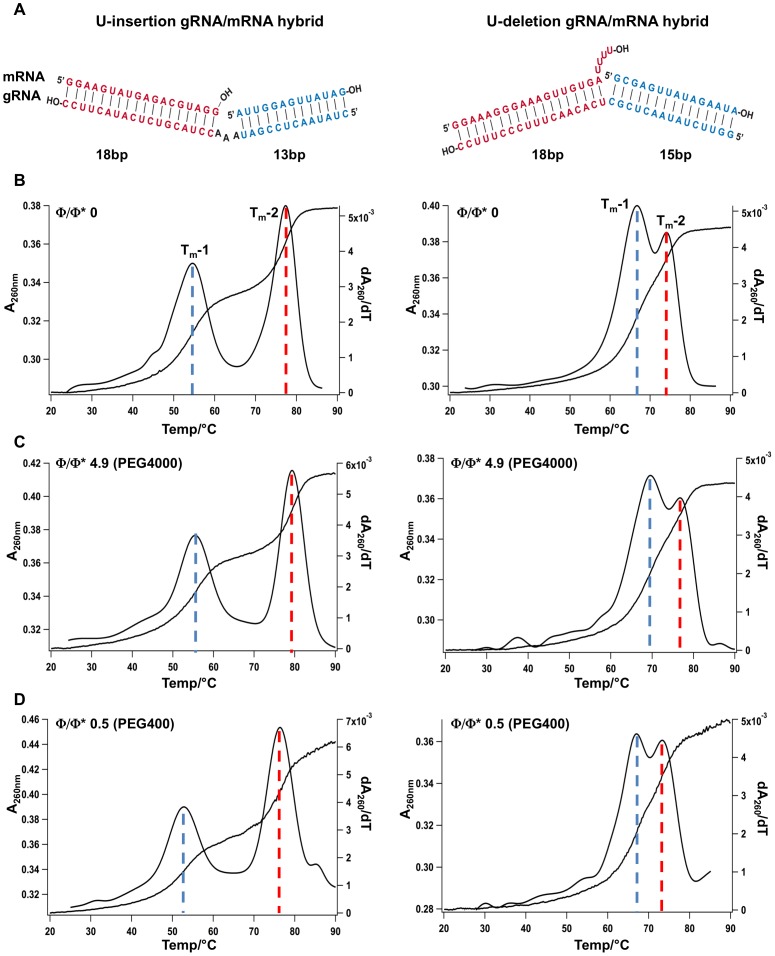
UV melting profiles of synthetic RNA editing substrate RNAs. (A) Schematic representation of the two model pre-mRNA/gRNA hybrid RNAs specific for a U-insertion (left) and a U-deletion (right) RNA editing reaction. Both RNAs consist of two helical domains shown in red and blue. (B) UV melting profiles (A_260_ = f(T)) and 1^st^ derivatives (dA_260_/dT = f(T)) of the two RNAs at dilute solvent conditions (Φ/Φ* = 0) in the absence of PEG. (C/D) UV melting profiles (A_260_ = f(T)) and 1^st^ derivatives (dA_260_/dT = f(T)) of the two RNAs at crowded (Φ/Φ* = 4.9) and semidilute (Φ/Φ* = 0.5) conditions. Dotted lines indicate the half maximal melting transitions of the two helical domains (blue: T_m_-1; red: T_m_-2).

Crowded intracellular environments are characterized by unique solvent properties such as a reduced number of free water molecules, which has been shown to affect the structure of nucleic acid molecules [23], [31]. Depending on the crowding reagent, stabilizing as well as destabilizing effects have been reported [17], [30]. U-insertion/deletion-type RNA editing is a RNA processing reaction that takes place within the mitochondria of kinetoplastid organisms. Despite the fact that mitochondria have been identified as the most severely crowded intracellular compartment [33], [34], editing has only been analyzed at highly dilute solvent conditions. This tempted us to test whether the structure of substrate gRNA/pre-mRNA hybrid RNAs of the editing reaction might be affected by crowded solvent conditions. gRNA/pre-mRNA hybrids adopt a three-helix-junction (THJ) geometry [46], [47], however, the molecules can be mimicked by hybridized, synthetic oligoribonucleotides that consist of only two helical elements. Fig. 2A shows two typical “pre-cleaved” gRNA/pre-mRNA hybrid RNAs specific for a U-insertion- and a U-deletion-type editing reaction. To analyze whether the two “model” editing RNAs become structurally altered at crowded solvent conditions we measured the temperature-dependent helix/coil transitions of the two RNAs in the presence of different crowding reagents at dilute (Φ<Φ*), semidilute (Φ≈Φ*) and crowded (Φ>Φ*) cosolute conditions. Fig. 2B shows representative UV melting curves of the two gRNA/pre-mRNA pairs in a dilute buffer (Φ/Φ* = 0). The two RNAs “melt” with two separate transitions (T_m_-1, T_m_-2), corresponding to the unfolding of the two RNA helices (Fig. 2A). The melting midpoints are at 54°C and 77°C for the U-insertion RNA and at 67°C and 74°C for the U-deletion substrate. Fig. 2C shows the same UV-melting profiles in the presence of 20% (w/v) PEG4000 *i.e.* at crowded solvent conditions 5-fold above the crossover polymer fraction (Φ/Φ* = 4.9). At these conditions, all melting transitions in both RNAs are shifted to higher temperatures with ΔT_m_s between 1.9°C and 3.3°C. This indicates a stabilization of the two RNA molecules. The stabilization calculates to a Gibb's free energy change (ΔΔG) of −31.4 kJ/mol for the U-insertion RNA and −33.7 kJ/mol for the U-deletion hybrid (all thermodynamic parameters are summarized in Tab. 2). Identical results were obtained at a Φ/Φ* ratio of 3 using 20% (w/v) PEG2000. The resulting ΔT_m_-values range from 1.7°C to 3.3°C equivalent to ΔΔGs of −29.4 kJ/mol (U-insertion substrate) and −31.6 kJ/mol (U-deletion RNA) (Tab. 2).

**Figure 3:**
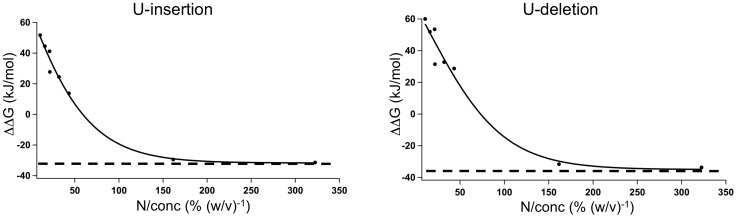
PEG-dependent RNA editing substrate stabilization. Gibb's free energy changes (ΔΔG) of the U-insertion (left) and U-deletion (right) pre-mRNA/gRNA hybrid RNAs in the presence of different polyethylene glycols. N - number of monomers/polymer (see Table 1); conc – PEG concentration in % (w/v). The dashed lines mark the maximal values of −33 kJ/mol for the U-insertion hybrid RNA and −38 kJ/mol for the U-deletion RNA.

By contrast, semidilute solvent conditions ranging from Φ/Φ* = 0.3–1.1 (PEG200, PEG300, PEG400) destabilized the two helical elements in both RNAs (as an example see Fig. 2D). The corresponding ΔT_m_s vary between −0.3°C to −7.3°C equivalent to ΔΔGs of 14 kJ/mol and 60 kJ/mol (Tab. 2). The destabilization is concentration-dependent: a doubling of the PEG concentration results in a 2- to 4-fold reduction of the T_m_-values. Furthermore, the destabilization is inversely correlated to the chain length of the PEG molecules. PEG200 is more “destabilizing” than PEG300 and PEG400 by about −2°C/100 Da. Fig. 3 summarizes the data by correlating the measured stability changes (ΔΔG) of the two RNAs to the number of monomers/polymer (N) and the concentration of the different PEGs (ΔΔG = f(N/conc). An increase per monomer stabilizes the U-insertion RNA by −1.2 kJ/mol/N/conc and the U-deletion RNA by −0.9 kJ/mol/N/conc. Maximal stabilization is achieved at −33 kJ/mol for the U-insertion pre-mRNA/gRNA hybrid and at −38 kJ/mol for the U-deletion RNA (Fig. 3).

### Editosome/RNA interaction at molecular crowding conditions

**Figure 4:**
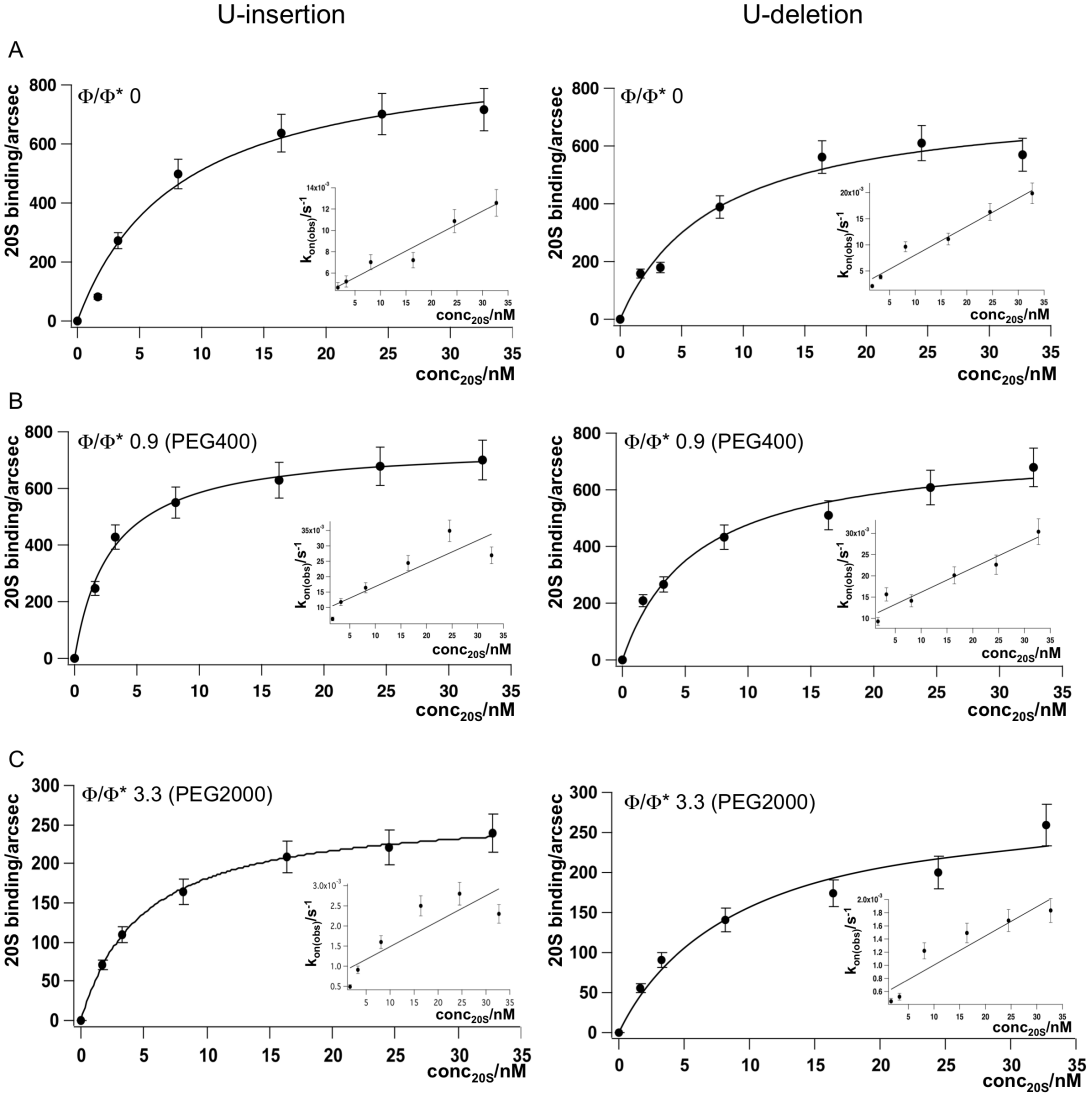
SPR-derived binding curves of 20 S editosomes to U-insertion (left) and U-deletion (right) pre-mRNA/gRNA hybrid RNAs. (A) Binding at dilute solvent conditions (Φ/Φ* = 0). (B) Binding at semidilute conditions (Φ/Φ* = 0.9) and (C) at crowded cosolute conditions (Φ/Φ* = 3.3). Inserts: Plots of k_on(obs)_ = f(conc_20S_) for the calculation of k_ass_ and k_diss_. Error bars are relative errors in percent.

Crowding reagents typically increase the viscosity of the solvent thereby influencing the thermodynamic and kinetic characteristics of biomolecular interactions [1], [2]. As a follow up of the above-described experiments we asked the question whether a volume-occupied/viscous solvent regimen affects the binding of editosomes to their substrate RNAs. In order to derive kinetic and thermodynamic data simultaneously, we monitored the editosome/RNA interaction in real time using a plasmon surface resonance (SPR)-based readout system. At dilute buffer conditions (Φ/Φ* = 0), the two reactants (20 S editosomes and gRNA/pre-mRNA hybrid RNAs) interact in a concentration-dependent fashion. The formation of the RNA/editosome complexes is complete within ≤5 min. Fig. 4A shows the corresponding binding curves for a U-insertion-type and a U-deletion-type gRNA/pre-mRNA hybrid RNA. The equilibrium dissociation constants (K_d_) for the binding reactions calculate to 6.4 nM (U-insertion RNA) and 6.6 nM (U-deletion RNA) indicating high affinity binding. The association- and dissociation rate constants (k_ass_ and k_diss_) range between 3.3−4.7×10^5^ M^−1^ s^−1^ and 2−3×10^−3^ s^−1^ and the calculated half-lives (t_1/2_) for the editosome/RNA complexes are 5.8 min (U-insertion RNA) and 3.9 min (U-deletion RNA). All binding characteristics are summarized in Table 3.

**Table 3:** Summary of binding data.

	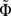 Φ*	k_ass_ (M^−1^s^−1^)	k_diss_ (s^−1^)	K_d_ (nM)	t_1/2_ (min)
**U-insertion**					
**w/o PEG**	0	3.3×10^5^	2.0×10^−3^	6.4	5.8
**25% (w/v) PEG400**	0.9	7.5×10^5^	9.0×10^−3^	12.0	1.3
**20% (w/v) PEG2000**	3.3	0.4×10^5^	0.6×10^−3^	13.4	20.6
**U-deletion**					
**w/o PEG**	0	4.7×10^5^	3.0×10^−3^	6.6	3.9
**25% (w/v) PEG400**	0.9	5.5×10^5^	10.0×10^−3^	18.0	1.2
**20% (w/v) PEG2000**	3.3	0.6×10^5^	0.9×10^−3^	12.6	13.6

k_ass_, k_diss_, K_d_ and t_1/2_ of 20 S editosomes to U-insertion and U-deletion mRNA/gRNA hybrid RNAs at semidilute (Φ/Φ* = 0.9) and crowded (Φ/Φ* = 3.3) solvent conditions.

By changing the solvent conditions to a semidilute regimen (Φ/Φ* = 0.9) the macroscopic Kd's for both RNA/editosome complexes increase to 12 nM (U-insertion) and 18 nM (U-deletion) (Fig. 4B and Table 3). The k_ass_- and k_diss_-rate constants increase up to 5-fold for the U-insertion hybrid and maximally 3-fold for the U-deletion RNA. As a consequence the half-lives (t_1/2_) of the RNA/editosome complexes decrease by a factor ≤5 (1.3 min for the U-insertion substrate; 1.2 min for the U-deletion RNA hybrid). By contrast, at crowded solvent conditions (Φ/Φ* = 3.3) the association and dissociation rate constants decrease 3 to 8-fold resulting in a roughly 4-fold longer half-live of the complexes (Fig. 4C and Table 3). Thus, the data demonstrate a vital difference between dilute, semidilute and crowded solvent conditions: the transition from a dilute to a semidilute regimen increases the rate constants for the formation and dissociation of the RNA/editosome complexes while at crowded conditions the rate constants decrease. This affects the half-lives of the complexes in opposite directions suggesting that the reaction switches from a slow, transition-state-limited association reaction in dilute and semidilute conditions to a fast, diffusion-limited reaction in crowded conditions [2].

### *In vitro* RNA editing at molecular crowding conditions

**Figure 5:**
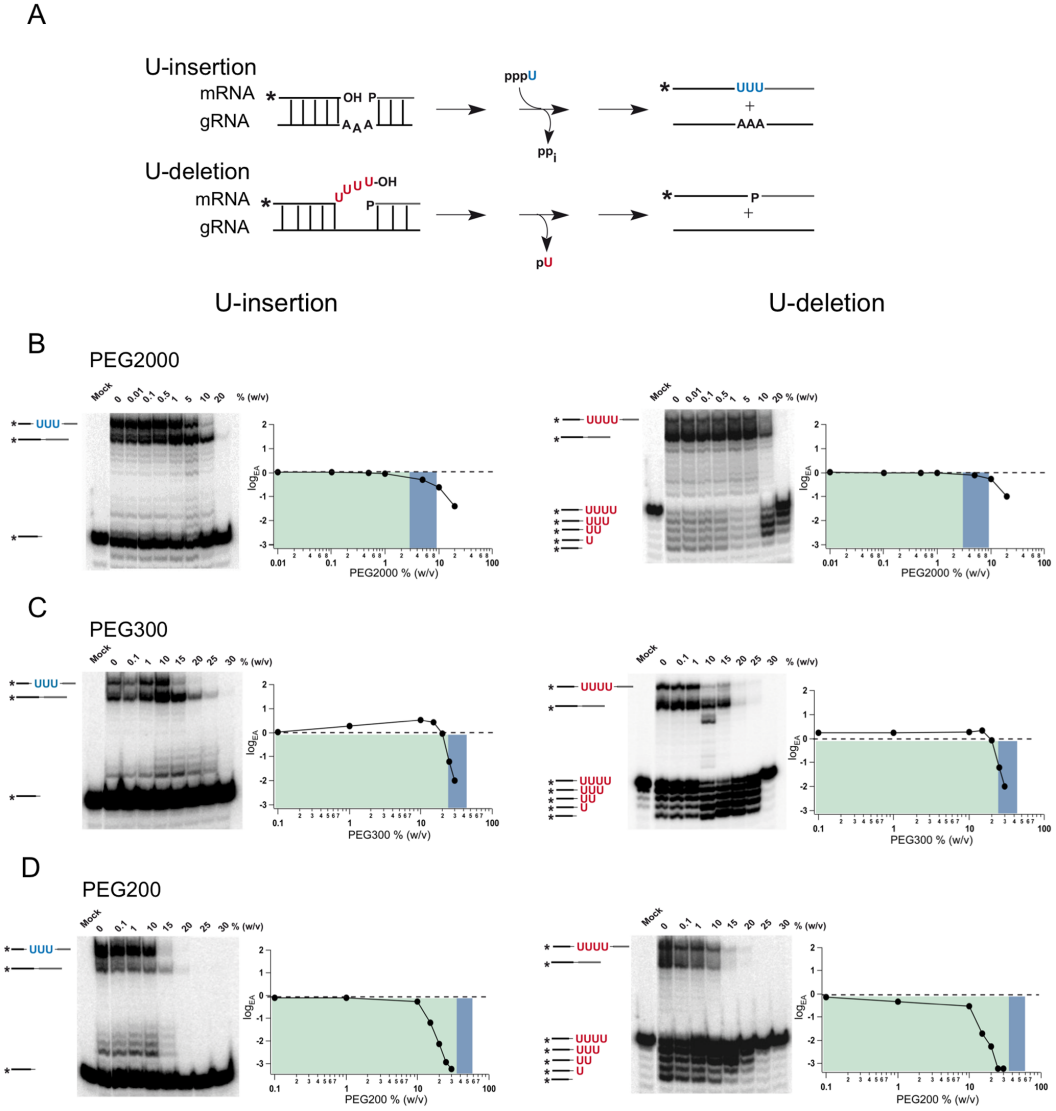
*In vitro* RNA editing at dilute, semidilute and crowded cosolute conditions. (A) Schematic representation of *in vitro* U-insertion and U-deletion editing reactions. Substrates in the assays are “precleaved” pre-mRNA/gRNA hybrid RNAs, which are converted to edited products either by the gRNA-dependent insertion of 3 U nucleotides (blue) or the deletion of 4 U's (red). Assays were performed at varying concentrations of PEG2000 (B), PEG300 (C) and PEG200 (D). RNA reactants, products and intermediates (sketched on the left of the autoradiographs) were resolved electrophoretically and densitometrically quantified. Editing activities (EA) were normalized to the EA in the absence of PEG (dashed line) and plotted as a function of the molecular crowder (MC) concentration: log_EA_ = f(logc_MC_). Green background: dilute solvent conditions; blue background: semidilute conditions; white background: crowded conditions. Mock: minus 20 S editosomes. (*) annotates the position of the radioactive label [^32^P].

In order to analyze whether the described structural, thermodynamic and kinetic consequences at crowded solvent conditions directly affect the catalytic conversion of a pre-edited mRNA into an edited reaction product, we measured the RNA editing activity of the two gRNA/pre-mRNA substrate RNAs directly. As before, the measurement was performed at different cosolute concentrations covering dilute, semidilute and concentrated solvent properties. The two model RNAs represent synthetic versions of the first editing site of the subunit 6 of the mitochondrial ATPase (A6) from *Trypanosoma brucei* [48], [49]. Depending on the presence of cognate gRNAs, either the site-specific insertion of 3 U nts into the pre-mRNA is monitored or alternatively the deletion of 4 U's from the pre-mRNA is analyzed (Fig. 5A). Fig. 5B shows representative examples of the two *in vitro* editing reactions in the absence/presence of PEG2000 as a cosolute. At dilute reaction conditions (Φ<Φ*) *in vitro* editing is not affected. When the PEG concentration increases to semidilute (Φ≈Φ*) and finally to crowded conditions (Φ>Φ*) the formation of the fully edited reaction products is completely stalled. At 20% (w/v) PEG2000 (Φ/Φ* = 3.3), insertion-type RNA editing is >50-fold reduced while deletional editing is decreased by a factor of >10. Identical results were gained with PEG4000 at Φ/Φ* = 4.9: both, U-insertion and U-deletion editing are inhibited between 50- to 100-fold. The U-insertion reaction is stalled at the TUTase and the mRNA ligation step, while the U-deletion reaction is inhibited at the exoUase and ligation reaction. Importantly, while the ligation reaction (in both cases) is inhibited to ≥95% (at the highest PEG concentration) the exoUase is not fully inhibited. Given the precursor/product relationship of the two reactions in the editing cycle [32] this suggests that the exoUase and the mRNA ligation activity can be inhibited independently.

**Figure 6:**
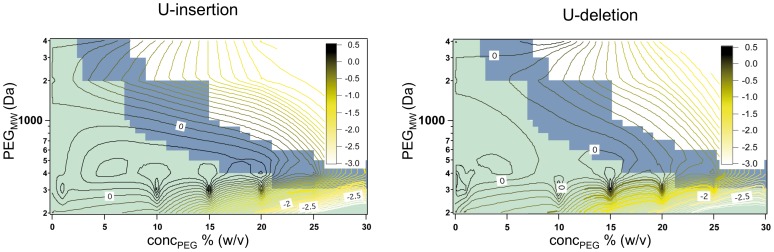
Contour plot correlating the U-insertion (left) and U-deletion (right) RNA editing activity to the polymer length and concentration of the different PEG molecules. Editing activities (EA) are normalized to the EA in the absence of PEG and are expressed as logEA on a scale of 0.5 to −3.0 (inserts). Green background: dilute solvent conditions; blue background: semidilute conditions; white background: crowded regimen.

Next to the two high molecular PEGs we analyzed the influence of three low molecular mass polyethylene glycols: PEG200, PEG300 and PEG400. Fig. 5C/D show representative examples of the analysis. At dilute reaction conditions with cosolute concentrations ≤10% (w/v) the two types' of editing are not affected: The three crowding reagents display editing activities identical to the situation in the absence of a cosolute (Φ/Φ* = 0). However, at PEG concentrations >10% (w/v) both, insertion and deletion editing are inhibited ≥100-fold identical to the situation at crowded conditions (Φ>Φ*) in the presence of high molecular mass PEGs. For PEG300 and PEG400 the inhibition takes place at or around the crossover concentration from a dilute to a semidilute regime, while for PEG200 the inhibition already occurs at dilute solvent conditions. Fig. 6 summarizes the concentration-dependence of the *in vitro* RNA editing activity for all PEG molecules tested.

A comparison of the inhibition profiles of the fully edited mRNA ligation products *versus* the non-edited ligation side-products showed for the U-insertion reaction that the fully edited mRNA is always inhibited at lower PEG concentrations when compared to the non-edited ligation product. By contrast, the U-deletion reaction showed an inverse behavior: the formation of non-edited side-product was always more sensitive to increased PEG concentrations in comparison to the fully edited mRNA (as an example see Fig. 5C). This supports a scenario in which the two RNAs are ligated by two different RNA ligase activities [50], [51].

Lastly, we analyzed whether other crowding reagents show similar characteristics as PEG and performed *in vitro* U-insertion editing reactions in the presence of high molecular mass cosolutes of different chemical origins: the high molecular mass polysaccharides Dextran150 and Ficoll400 as well as bovine serum albumin (BSA) as a protein-type crowding reagent. All three compounds were analyzed at concentrations up to 10% (w/v) (data not shown). At dilute and semidilute conditions none of the reagents showed any effect. However, at crowded cosolute conditions inhibition of editing was identified identical to the situation with PEG. This demonstrates that the described inhibitory effect is independent of the chemical signature of the crowding reagent.

## Discussion

The U nucleotide-specific insertion/deletion-type RNA editing reaction in kinetoplastid organisms is a mitochondria-specific biochemical process and as such it must be tolerant to the highly crowded environment within the organelle [52]. However, the processing reaction has so far only been analyzed at dilute, buffered solvent conditions, which fail to measure the contribution of other factors to RNA stability and functionality, especially the excluded volume and hydration effects triggered by chemically inert cosolutes. Here, we investigated the structures of two synthetic model gRNA/pre-mRNA editing substrates, their interaction with 20S editosomes and their *in vitro* RNA editing activity at dilute, semidilute and crowded cosolute conditions. We identified that both, high and low molecular mass crowding reagents (PEGs) affect the structure of the two RNAs. Low molecular PEGs (PEG200, PEG300, PEG400) have a destabilizing effect at semidilute conditions in the range of 60 kJ/mol, while high molecular mass PEGs (PEG2000, PEG4000) at crowded conditions stabilize the two RNAs by about −30 kJ/mol. The stabilization correlates with the polymer size and concentration of the different PEGs with a value of about −1.0 kJ/mol/N/conc. In line with published data, the stabilization is most likely explained by the volume exclusion effect, while the destabilization is caused by a decrease in water activity [18], [30]. Importantly, both phenomena are able to inhibit RNA editing *in vitro* (see below).

In order to initiate the processing reaction, pre-edited mRNAs and guide RNAs have to bind to the single substrate RNA binding site of the editing machinery [37]. RNA binding to editosomes has been analyzed before at dilute solvent conditions and was characterized as a high affinity interaction with K_d_'s in the nanomolar range [36], [37]. Here we measured the RNA-binding capacity of editosomes in real time using semidilute and crowded solvent conditions. In both cases, the macroscopic K_d_'s decreased only by a factor ≤3. Thus, even at crowded cosolute conditions editosomes and mRNA/gRNA hybrid RNAs can interact with high affinity. However, a comparison of the rate constants for the association and dissociation of the editosome/RNA complexes identified a crucial difference between the two solvent settings: While the k_ass_- and k_diss_-values increased at semidilute conditions, the two constants decreased in crowded conditions. Similarly, while the half-lives of the complexes decreased at semidilute conditions, they increased at crowded conditions. This suggests that the processing reaction converts from a slow, transition-state-limited association reaction in dilute and semidilute conditions to a fast, diffusion-limited reaction in crowded conditions [2]. As a consequence, both subtypes of the editing reaction (U-insertion and U-deletion) are inhibited. For the two tested high molecular mass PEGs (PEG2000, PEG4000), the inhibition occurs exactly at the crossover concentration from a semidilute to crowded solvent regime suggesting volume exclusion as the dominant factor. The low molecular PEGs inhibit the reaction at lower concentrations (PEG400>PEG300>PEG200) perhaps as a result of a combination of hydration and excluded volume effects.

The reaction is inhibited at every step of the enzymatic reaction cycle (TUTase, exoUase, RNA ligation). This classifies the cosolute-induced inhibition as a general phenomenon, which is further supported by the fact that other crowding reagents (Ficoll400, Dextran150, BSA) inhibit the reaction with similar characteristics. A comparison of the inhibition profiles of the fully edited reaction products *versus* the non-edited side products demonstrated that the two ligase reactions are inhibited at different cosolute concentrations. This suggests the presence of two different enzymes in line with the fact that 20S editosomes harbor two RNA ligases (TbMP48/REL2 and TbMP52/REL1) [32]. This is further supported by the structural observation that 20S editosomes consist of two prominent globular subdomains [36], likely representing the individual subdomains of the U-insertion and U-deletion reactions [35].

Whether the inhibition is a direct consequence of the structural stabilization of the RNA substrate molecules or a result of the decreased k_ass_- and k_diss_-values (or both) cannot be deduced from the data presented here. However, the sensitivity of the *in vitro* assay to crowded cosolute conditions demonstrates that the assay does not recapitulate a central aspect of the *in vivo* situation: editing must be conducted in the densely volume-occupied environment inside the mitochondria. Though the *in vitro* assay has been instrumental in elucidating the basic aspects of the editing reaction cycle, clearly, the *in vivo* reaction cannot rely on a diffusion-limited, collision-based mechanism (which might also explain other inconsistencies of the editing *in vitro* assay). Our data advocate a scenario in which editing *in vivo* is conducted by non-diffusional means perhaps through the coupling of substrate RNAs by physically interfacing the participating machineries downstream and upstream of the editing reaction. Precedence for such a situation can be found in the physical and functional tethering of the gene expression pathway in eukaryotes. The entire process (transcription, pre-mRNA processing, cytoplasmic export, translation) is conducted by several macromolecular, multi-component complexes, which act as an extensively coupled network that executes the individual biochemical reactions in a highly coordinated fashion [53], [54]. This involves a “handover” or “channeling” of substrate RNAs from one complex to the next instead of relying on free aqueous-phase diffusion. Evidence for a possible coupling of editing to down- and upstream processes can be found in the literature. For instance, Aphasizheva et al., 2011 [55] have shown that mitochondrial mRNAs, gRNAs and editosomes interact with the mitochondrial translation machinery: pre-edited mRNAs, gRNAs and editosomes bind predominantly to the large subunit of the ribosome and fully edited, A/U-tailed mRNAs associate with the small ribosomal subunit. This suggests a functional tethering of editing, polyadenylation and protein biosynthesis. The interaction likely involves one or more pentatricopeptide repeat-type (PPR) proteins, which have been shown to bind to ribosomes and have been implicated in the stabilization of rRNAs [55], [56]. A potential coupling of transcription and editing can be deduced from the work of Read et al., 1992 [57]. They demonstrated polycistronic transcription of mitochondrial genes in trypanosomes and verified that RNA editing can precede processing and polyadenylation of the primary transcript.

**Figure 7:**
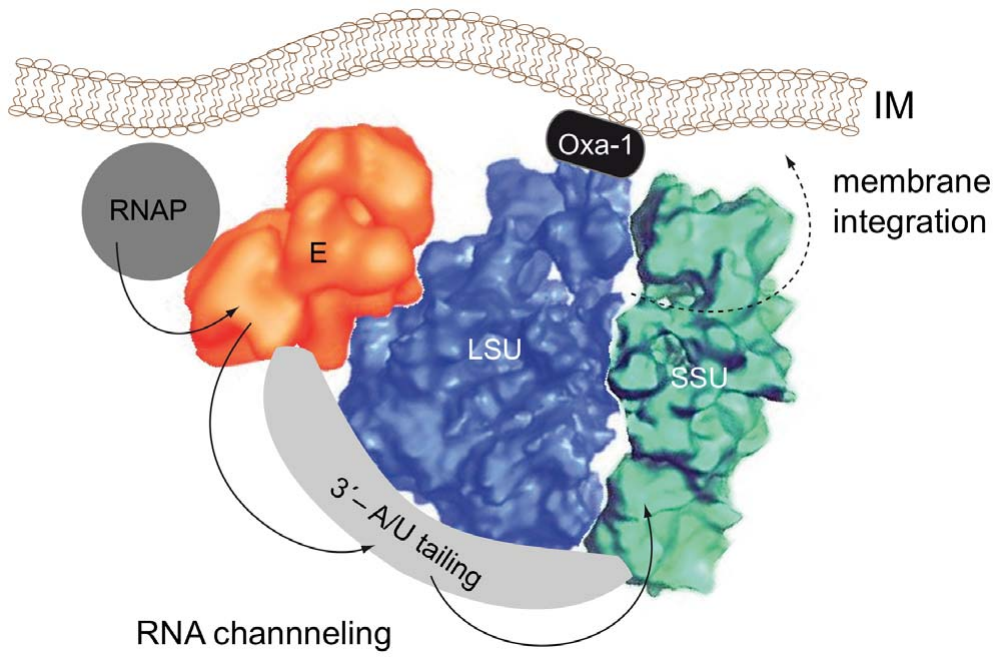
Tethering of mitochondrial transcription, RNA editing, 3′-end processing and translation. Schematic representation of a coupled, inner membrane-associated (IM) assembly of 20 S editosomes (E) [36] with mitochondrial ribosomes (SSU/LSU) [61] and the mitochondrial transcription machinery (RNAP) [62]. The different macromolecular complexes interface physically in order to foster the channeling of substrate RNAs (arrows) from one machinery to the next thereby side-stepping free aqueous-phase diffusion. A coupling of polycistronic gRNA transcripts with RNA editing complexes has been demonstrated by Grams et al., 2000 [63]. Read et al., 1992 [57] verified polycistronic transcription of mitochondrial genes and showed that RNA editing can precede processing and polyadenylation. Edited mRNAs are polyadenylated by the extension of A/U-heteropolymers, which is catalyzed by the poly(A) polymerase KPAP and the terminal uridyltransferase RET1. The reaction is coordinated by the pentatricopeptide-repeat (PPR) proteins KPAF1 and KPAF2 [55]. Fully edited, A/U-tailed mRNAs have been shown to preferentially interact with the SSU, while pre-edited mRNAs, gRNAs and 20 S editosomes have been shown to bind to the LSU [55]. Mitochondrial ribosomes associate with membranes either through electrostatic interactions [59] or *via* specific membrane-associated protein(s) such as Oxa-1 [60]. The dashed arrow annotates the membrane-integration of the translation products.

Finally, another factor possibly contributes to the non-diffusional characteristics of the editing reaction *in vivo*. The physical interaction of editosomes with the mitochondrial translation machinery might position the processing machinery in close proximity to the inner mitochondrial membrane (IM). A membrane-association of mitochondrial ribosomes is presumably essential in order to couple the synthesis of hydrophobic membrane proteins to the membrane integration process [58]. Mitochondrial ribosomes have been shown to associate with membranes either through electrostatic interactions [59] or *via* specific, membrane-associated protein(s) [60]. Since the majority of genes that require RNA editing are components of membrane-associated, respiratory complexes (NADH-ubiquinone oxidoreductase - complex I, cytochrome bc1 - complex III, cytochrome oxidase - complex IV and ATP synthase - complex V), fixing the editosome (indirectly) to the inner mitochondrial membrane should increase the local concentration of all reaction partners and substrate molecules thereby generating a “diffusion-independent” scenario (Fig. 7). Although a membrane-association of editosomes has not been documented today, this is likely due to the fact that the standard enrichment protocol for 20 S editosomes involves a detergent extraction step [39]. In conclusion, we propose that mitochondrial transcription, RNA editing, 3′-end processing and mitochondrial translation occur in close physical association in African trypanosomes. The individual machineries possibly interact in a coordinate, membrane-associated form thereby side-stepping diffusional processes, which is not mimicked in the current *in vitro* RNA editing assay.
